# The inhibitory effects of extracellular ATP on the growth of nasopharyngeal carcinoma cells via P2Y2 receptor and osteopontin

**DOI:** 10.1186/1756-9966-33-53

**Published:** 2014-06-24

**Authors:** Guang Yang, Shenghong Zhang, Yanling Zhang, Qiming Zhou, Sheng Peng, Tao Zhang, Changfu Yang, Zhenyu Zhu, Fujun Zhang

**Affiliations:** 1 State Key Laboratory of Oncology in South China, Department of Imaging and Interventional Radiology, Cancer Center, Sun Yat-sen University, Guangzhou Guangdong 510060, China; 2 Division of Gastroenterology, The First Affiliated Hospital, Sun Yat-sen University, Guangzhou, China; 3 School of Biotechnology, Southern Medical University, Guangzhou 510515, China; 4 Department of Biochemistry and Molecular Biology, Zhongshan Medical College, Sun Yat-sen University, Guangzhou, China

**Keywords:** Nasopharyngeal carcinoma, Extracellular ATP, Osteopontin, P2Y2 receptor

## Abstract

**Background:**

Nasopharyngeal carcinoma (NPC) is a common malignant tumor observed in the populations of southern China and Southeast Asia. However, little is known about the effects of purinergic signal on the behavior of NPC cells. This study analyzed the effects of ATP on the growth and migration of NPC cells, and further investigated the potential mechanisms during the effects.

**Methods:**

Cell viability was estimated by MTT assay. Transwell assay was utilized to assess the motility of NPC cells. Cell cycle and apoptosis were detected by flow cytometry analysis. Changes in OPN, P2Y2 and p65 expression were assessed by western blotting analysis or immunofluorescence. The effects of ATP and P2Y2 on promoter activity of OPN were analyzed by luciferase activity assay. The binding of p65 to the promoter region of OPN was examined by ChIP assay.

**Results:**

An MTT assay indicated that ATP inhibited the proliferation of NPC cells in time- and dose-dependent manners, and a Transwell assay showed that extracellular ATP inhibited the motility of NPC cells. We further investigated the potential mechanisms involved in the inhibitory effect of extracellular ATP on the growth of NPC cells and found that extracellular ATP could reduce Bcl-2 and p-AKT levels while elevating Bax and cleaved caspase-3 levels in NPC cells. Decreased levels of p65 and osteopontin were also detected in the ATP-treated NPC cells. We demonstrated that extracellular ATP inhibited the growth of NPC cells via p65 and osteopontin and verified that P2Y2 overexpression elevated the inhibitory effect of extracellular ATP on the proliferation of NPC cells. Moreover, a dual luciferase reporter assay showed that the level of osteopontin transcription was inhibited by extracellular ATP and P2Y2. ATP decreased the binding of p65 to potential sites in the OPN promoter region in NPC cells.

**Conclusion:**

This study indicated that extracellular ATP inhibited the growth of NPC cells via P2Y2, p65 and OPN. ATP could be a promising agent serving as an adjuvant in the treatment of NPC.

## Introduction

Accumulating evidence has associated purinergic signals, which are induced by extracellular nucleotides, to the processes of several diseases [1]. Extracellular nucleotides, particularly ATP, are important transmitters that mediate various biological effects via purinergic receptors (P2-receptors) in many cell types [2], and several studies have found that ATP can inhibit tumor growth [3-6]. P2 receptors are subclassified into two main types: P2X and P2Y receptors [7]. P2X receptors are ligand-gated ion channels that are activated by extracellular ATP to elicit a flow of cations [2], seven of which, P2X1 to P2X7, have been cloned [8]. The metabotropic P2Y receptors belonging to the G-protein-coupled receptor (GPCR) family play important roles in several signaling pathways, and eight P2Y receptors have been cloned and identified as GPCRs in mammals [2,9]. Although P2Y receptors are distributed in a wide range of normal tissues, P2X receptors are mainly expressed in the nervous system, platelets, and smooth muscle cells (SMCs) [10,11]. P2Y2 has often been reported to be a functional receptor that transduces several biological signals induced by ATP and UTP, studies largely conducted in normal cells, such as epithelial cells, smooth muscle cells, leukocytes, and nerve cells. It has also been reported that P2Y2 activates nerve growth factor signaling to enhance neuronal differentiation and is also involved in phagocytic clearance [12-15]. However, the role of P2Y2 in tumor cells remains poorly understood, though some reports have described the possible role of P2Y2 in effects of extracellular nucleotides on tumor cells [4]. Osteopontin (OPN) is a secreted arginine-glycine-aspartic acid (RGD)-containing phosphoprotein with a thrombin cleavage site. By binding to several integrins and CD44 variants, OPN plays an important role in tumorigenesis, tumor invasion, tumor growth, and metastasis in many types of cancers [16-18]. OPN has been shown to promote cell survival through the inhibition of apoptosis, and OPN downregulation decreases the motility and invasiveness of tumor cells [19,20]. Although it has been reported that extracellular nucleotides induce OPN expression in arterial SMCs [21], their effect on OPN expression in tumor cells has not been examined.

Purinergic signaling has thus far not been investigated in NPC cells, and the effect of extracellular ATP on tumor cell OPN levels is unclear. Therefore, the effects of ATP on NPC cell apoptosis, cell cycle arrest, and cell migration were investigated in the present study, and we also explored whether the effects were caused through P2Y2 and OPN.

## Materials and methods

### Materials

ATP was purchased from Amersco (Solon, Ohio, USA), prepared in water, and stored in aliquots of an appropriate volume at -20°C until use. The antibodies used were anti-OPN (Sigma, USA), anti-P2Y2 (Santa Cruz, USA), and anti-cleaved caspase-3 (Cell Signal Technology, USA); other antibodies were purchased from Beyotime (Nantong, China).

### Cell culture and transfection

The cell lines 5-8 F and CNE-2 were gifts from Dr. Shan Jiang, State Key Laboratory of Oncology in Southern China, SunYat-Sen University, Guangzhou, P. R. China. The cells were cultured in DMEM supplemented with heat-inactivated fetal calf serum and penicillin (100 U/ml)/streptomycin (100 mg/mL) in 5% CO_2_ at 37°C. The transfection of plasmids and siRNA was performed using Lipofectamine 2000 (Invitrogen, USA) according to the manufacturer’s instructions.

### MTT assay

The cell lines were seeded in 96-well plates at a density of 5000 cells per well in a volume of 150 μL of culture medium per well. After 24 h, ATP was added to the wells at different concentrations in triplicate. The plates were incubated at 37°C in 5% CO_2_ for 24 or 48 h. If a transfection was performed, ATP was added to the wells after the transfection for 24 h. A 20-μL sample of MTT solution (5 g/L, dissolved in PBS) was added to each well, and the plates were incubated at 37°C for an additional 4 h. The supernatant was discarded, and l50 μL of DMSO was added to dissolve the formazan product. The absorbance values at 570 nm (A_570_) were determined using a multi-well plate reader (Tecan, Maennedorf, Switzerland).

### Measurement of intracellular calcium

The change of intracellular Ca^2+^ concentration in the cultured 5-8 F and CNE2 cells was measured using the fluorescence probe fluo-3 with acetoxymethyl ester. The two cell lines were grown in 96-well plates (black well, clear bottom; Greiner Bio One) until confluence. The dyes were loaded into the cells by adding 5 μM fluo-3 AM from 1 mM stock in DMSO to a bath solution containing the following: 20 mM HEPES, 140 mM NaCl, 5 mM KCl, 1 mM CaCl_2_, 1 mM, K_2_HPO_4_, 1 mM MgCl_2_ and 5 mM glucose with the pH adjusted to 7.4. The cells were incubated for 40 min at 37°C in the dark. After loading, the cells were subsequently washed three times in bathing solution, and then, 100 μl of bathing solution was added to each well. The plates were then placed in a multifunctional multi-well plate reader (Genios Plus, Tecan). The fluorescence density was measured after the addition of 100 μl of control buffer or 100 μl of the indicated concentration of ATP in bath solution with or without receptor antagonists (PPADS, suramin). The absorption wavelength was 535 nm, and the excitation wavelength was 485 nm. The fluorescence of intracellular Ca^2+^ was also observed under inverted fluorescence microscopy.

### siRNA sequences and plasmid construction

The coding sequences of P2Y2 and OPN were amplified from CNE-2 cells, and each was cloned into pcDNA3.1. The siRNA used for human OPN knockdown was a mixture of 5′-GUGGGAAGGACAGUUAUGATT-3′ and 5′-GUCUCACCAUUCUGAUGAATT-3′. The siRNA control sequence was 5′-ACGCATGCATGCTTGCTTT-3′. The plasmid used for p65 knockdown was a gift from Dr Yang Zhang (Department of Biochemistry and Molecular Biology, Zhongshan School of Medicine, Sun Yat-Sen University, China) and was based on pSilence2.0-U6, with the following sequence: 5′-CGCTGCAGTTTGATGATGAATTCAAGAGATTCATCATCAAACTGCAGCTTTTTTG-3′. P2Y2-shRNA expression plasmids were constructed based on plasmid pcDNA3.1(+) (Invitrogen, USA) according to a previous report [22]. EGFP was amplified by PCR from pEGFP-N1 (Clontech, USA), and the human U6 promoter was obtained from the human genome. The EGFP fragment was inserted between the KpnI and Bam HI sites of pcDNA3.1(+), and the U6 promoter was inserted into the Bam HI and Eco RI sites of pcDNA3.1(+). The siRNA sequence targeting the human P2Y2 transcript and the control sequence were as follows:

Control (NC), 5′-AATTCTTCTCCGAACGTGTCACGTTTCAAGAGAACGTGACACGTTCGGAGAATTTTTT-3′;

P2Y2-shRNA, 5′-AATTCCGCCATCAACATGGCCTACTTCAAGAGAGTAGGCCATGTTGATGGCGTTTTTT-3′.

### Flow cytometry analysis

A cell cycle analysis was performed by flow cytometry. DNA labeling was performed using the Cycletest Plus DNA Reagent kit (BD Biosciences Pharmingen, USA), and the samples were analyzed using a flow cytometer (Beckman Counter, USA). For the detection of apoptotic cells, labeling tests involving both propidium iodide (PI) and annexin-V were performed using an annexin-V staining kit (Invitrogen, USA) according to the manufacturer’s instructions.

### Transwell assay

Cell migration assays were performed using the Transwell (Costar, USA) system, which allows cells to migrate through 8-μm pore polycarbonate membranes. ATP (100 μM) was included or omitted in the medium in both the upper and lower compartments of the chambers. The chambers were incubated for 24 h at 37°C in humidified 5% CO_2_ air. The membranes were then washed with PBS, and the cells were fixed with cold methanol for 15 min and stained with crystal violet. The cells beneath the membrane were counted in 5 high-power microscopic fields. Each experiment was performed using three Transwell chambers and repeated three times.

### Immunofluorescence

Cells were treated with ATP for 36 h, washed with PBS, and fixed for 20 min at room temperature with 4% paraformaldehyde. The cells were washed again with PBS and permeabilized with Triton X-100 for 10 min at room temperature; the cells were then washed with PBS, incubated with 3% BSA to block non-specific binding sites, and incubated with primary antibodies at 4°C for 15 h. The cells were washed three times with PBS, incubated with the secondary antibody for 1 h at room temperature, and observed using an inverted fluorescence microscope after washing three times with PBS.

### Western blot analysis

Cells were exposed to various experimental conditions for the indicated times before being harvested and lysed for protein extraction. The protein concentration was determined using the Bio-Rad protein assay kit (Bio-Rad, P. R. China). The blots were visualized using an enhanced chemiluminescence detection system (Amersham, Pittsburgh, USA). β-actin was used as a loading control.

### Luciferase reporter assay

5-8 F and CNE-2 cells were seeded at 5 × 10^3^ per well in 96-well plates the day before transfection. A 683-bp promoter region (-365 to +318) of OPN was inserted into the pGL3-Basic luciferase reporter vector (Promega, USA). The cells were co-transfected with 0.1 μg of firefly luciferase reporter construct, 0.01 μg of pRL-TK Renilla luciferase reporter plasmid (Promega, USA), and the pcDNA3.1-P2Y2 vector using Lipofectamine 2000 (Invitrogen, USA). The luciferase activity was examined using a dual-luciferase reporter assay system (Promega, USA) according to the manufacturer’s instructions, and the signal was normalized to the internal Renilla control for the transfection efficiency.

### Chromatin immunoprecipitation

5-8 F and CNE-2 cells treated with or without ATP were utilized for chromatin immunoprecipitation using the EZ ChIP kit (Millipore, USA) according to the manufacturer’s instructions. After elution and purification, the recovered immunoprecipitated DNA samples were used for PCR with primers 5′-CAGTTGCAGCCTTCTCAGC-3′ (forward) and 5′-CCTTTGTTCCACAGGAGACC-3′ (reverse) to amplify a 201-bp segment of the OPN promoter containing the potential p65 binding sites. The PCR products were analyzed by agarose gel electrophoresis.

### Statistical analysis

A statistical analysis was performed using the unpaired Student’s *t*-test. P values < 0.05 were considered statistically significant.

## Results

### Effects of extracellular ATP on the proliferation and migration of NPC cell lines

**Figure 1:**
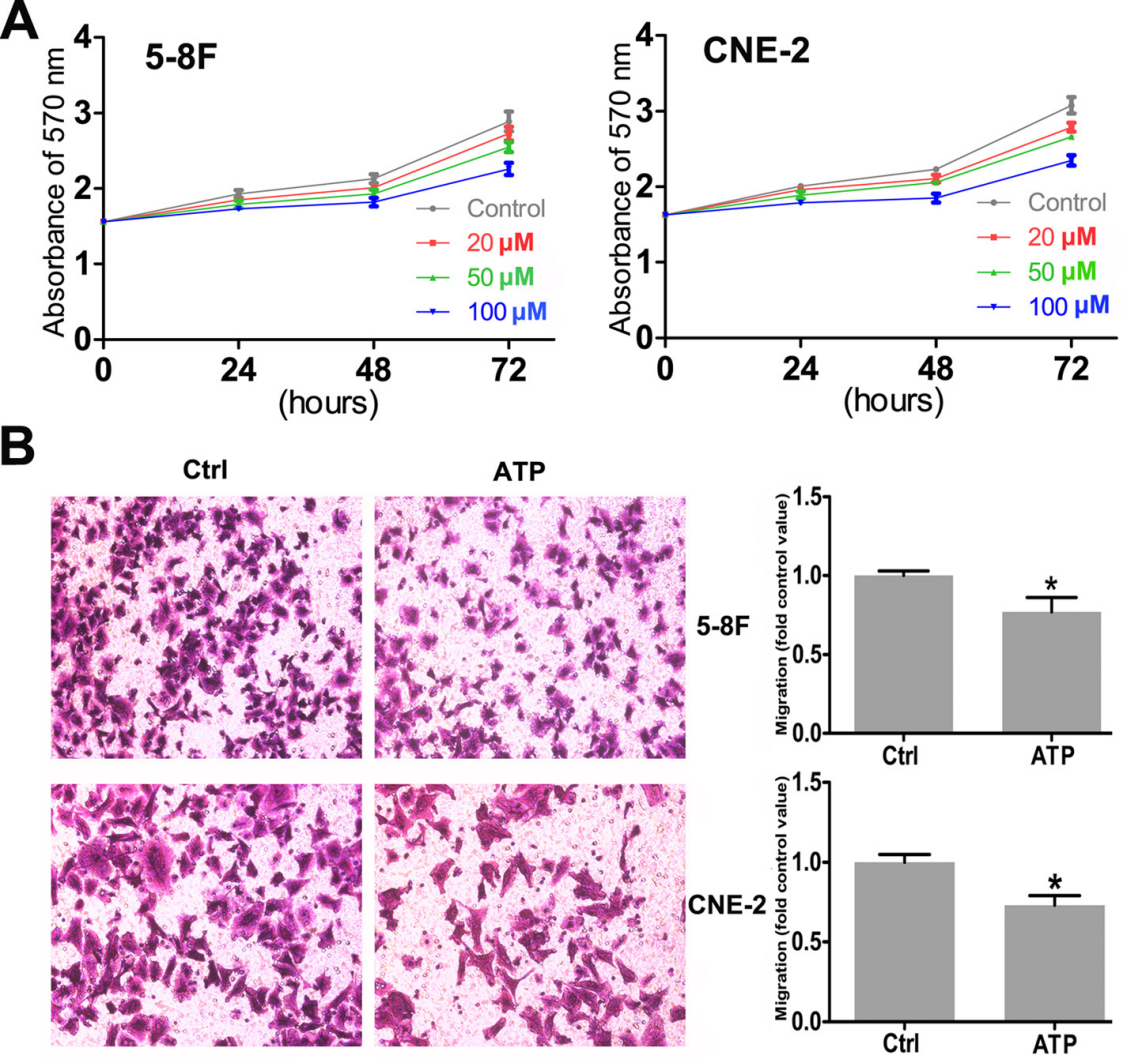
**Effects of ATP on the growth and migration of nasopharyngeal carcinoma cell lines. A**: Dose-dependent and time-dependent effects of ATP on 5-8 F and CNE-2 cell growth after incubation with ATP. The data represent the means ± SD of 3 independent experiments performed in quadruplicate. Statistical significance between the control and treated conditions: P < 0.05 for 50–100 μM of 24 h and 20 μM of 48 h and 72 h; P < 0.01 for other time points. **B**: ATP inhibited the migration of 5-8 F and CNE-2 cells. NPC cell migration was evaluated after incubation with ATP (10 μM) for 24 h. The data represent means ± SD of relative migration vs. control from 3 experiments. Statistical significance between the control and treated conditions: *P < 0.05.

The viability of the two cell lines was measured by an MTT assay after treatment with ATP for 24, 48, and 72 h. The growth of 5-8 F and CNE-2 cells was inhibited by treatment with ATP (20 μM, 50 μM, or 100 μM) in a dose- and time-dependent manner, with a striking effect after 48 h of treatment (Figure 1A). The effect of ATP (10 μM) on the migration of cultured 5-8 F and CNE-2 cells was measured using the Transwell system by counting the cells that migrated below the membrane (Figure 1B). We found the notable inhibition by ATP on the motility of the two NPC cell lines: after treatment with 10 μM ATP for 24 h, the motility of the 5-8 F and CNE-2 cells was 23.3% and 27.1% lower than the control groups.

### ATP induces apoptosis and S-phase arrest in NPC cells

**Figure 2:**
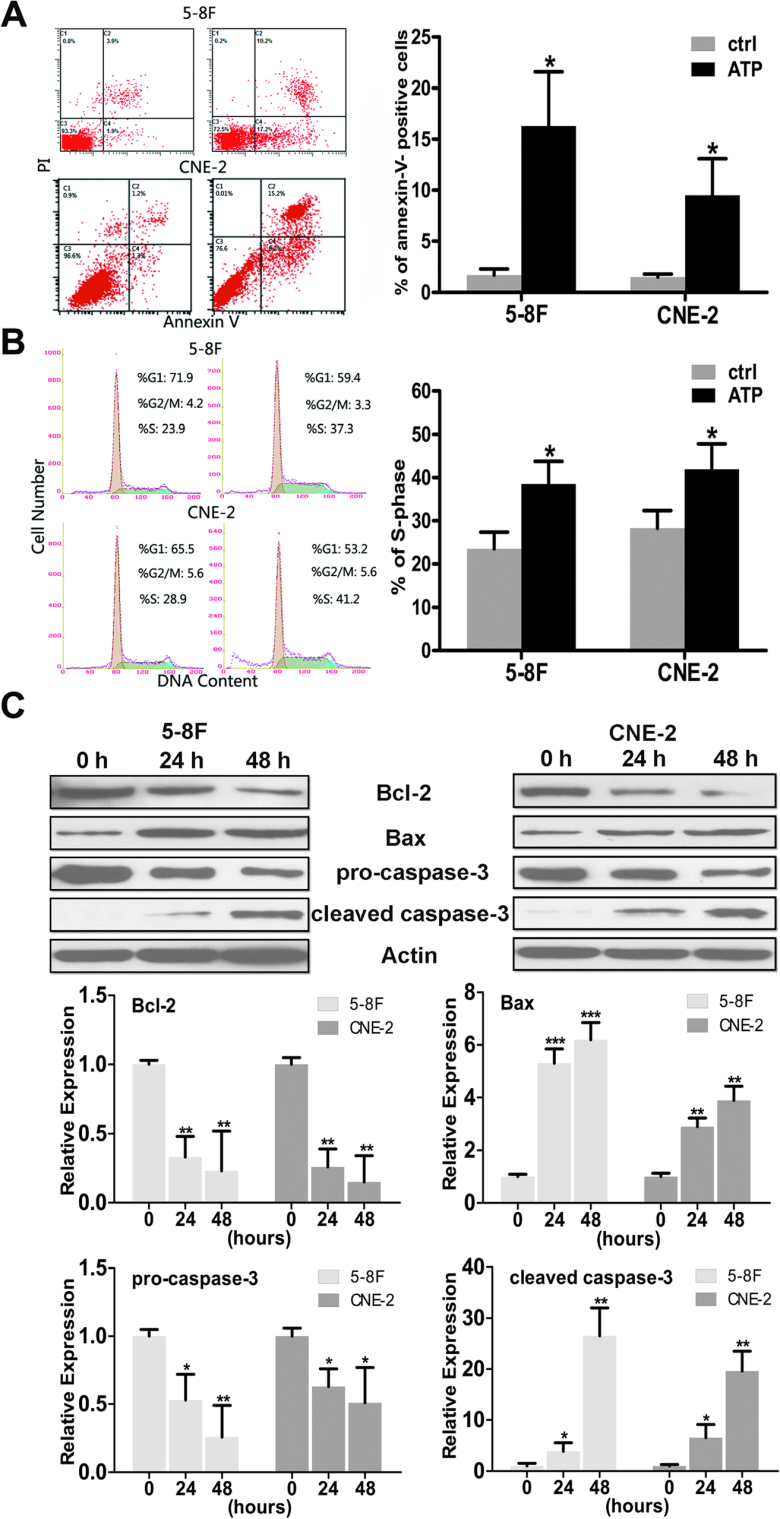
**Flow cytometry analysis of apoptosis and the cell cycle. A**: ATP induced apoptosis in NPC cells. 5-8 F and CNE-2 cells were treated with ATP for 48 h at a concentration of 100 μM; a flow cytometry analysis (staining with propidium iodide and annexin V-FITC) for cell death was performed. The lower left section of each graph represents the viable cells, the lower right section represents the apoptotic cells, and the upper right section represents the necrotic and late apoptotic cells. The total percentage of the counts in the lower right and upper right sections was calculated. **B**: ATP induced S phase arrest in NPC cells. 5-8 F and CNE-2 cells were treated with ATP for 24 h at a concentration of 100 μM, and the cells were subjected to a flow cytometry analysis. ATP showed a significant influence on the distribution of the cell cycle in 5-8 F and CNE-2 cells. **C**: The level of Bax was elevated, whereas Bcl-2 decreased in the ATP-treated NPC cells; the cleaved band of caspase-3 indicated that apoptosis had occurred in the ATP-treated NPC cells. Statistical significance between the control and treated conditions: *P < 0.05, **P < 0.01 and ***P < 0.001.

A flow cytometry analysis with propidium iodide (PI) and annexin V staining was performed to confirm apoptosis. As shown in Figure 2A, there was a significant increase in apoptotic cells after 48 h of incubation. Treatment with 100 μM ATP resulted in increased levels of annexin V-positive and PI-positive cell fractions of 5-8 F and CNE-2 cells (5.8% versus 27.4%; 2.5% versus 23.4%), indicating that ATP induced apoptosis in NPC cells. The cell cycle was analyzed to assess whether the growth inhibitory effect of extracellular ATP on NPC cells was mediated by cell cycle arrest. As shown in Figure 2B, incubating with ATP (100 μM) for 24 h caused a significant increase in the proportion of 5-8 F and CNE-2 cells in S phase (23.9% versus 37.3%; 28.9% versus 41.2%). The levels of proteins associated cell apoptosis, such as Bax, Bcl-2, and cleaved caspase-3, were also detected after treated with 100 μM ATP for the indicated time. As shown in Figure 2C, the level of Bcl-2 decreased, whereas Bax increased; an obvious cleaved caspase-3 band was also observed in the NPC cell lines.

### Intracellular calcium levels are changed in response to ATP

**Figure 3:**
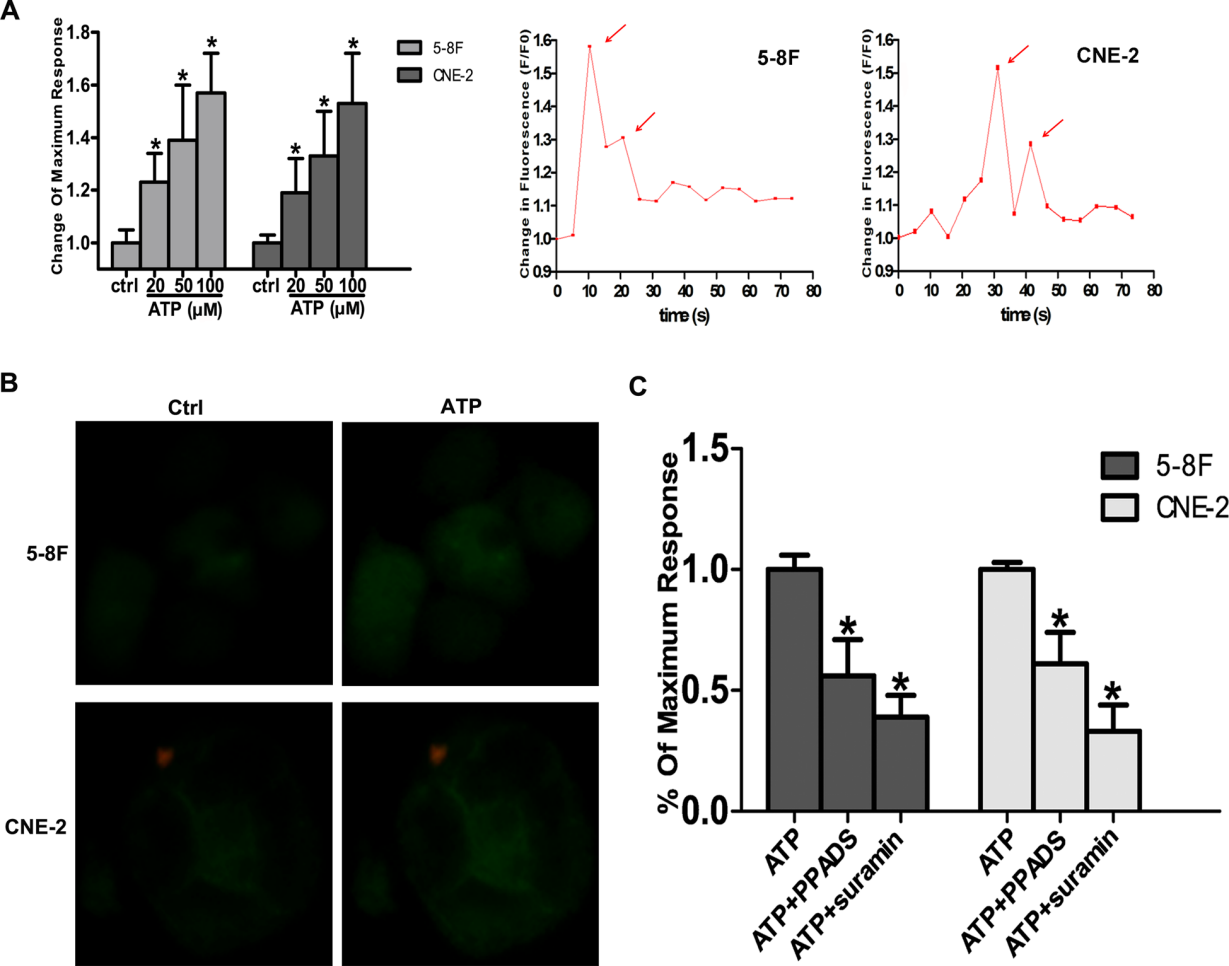
**The response of intracellular Ca**^**2+ **^**levels after ATP stimulation with or without receptor antagonists (PPADS, suramin). A**: The representative response curves of intracellular calcium level after ATP treatment. **B**: The representative fluorescence changes of intracellular Ca^2+^ obtained under inverted fluorescence microscopy. **C**: ATP induced a response of intracellular calcium level in dose dependent manner, and the receptor antagonists (PPADS, suramin) reduced the response after ATP treatment.

The addition of ATP increased the calcium concentration in 5-8 F and CNE-2 cells in a dose-dependent manner. An initial transient [Ca^2+^]i peak was followed by a secondary reduced phase. The biphasic response to stimulation with ATP implies the existence of metabotropic P2Y-receptors. The first response suggested the mobilization of Ca^2+^ from intracellular stores. The second peak is due to the calcium-mediated calcium influx across the cell membrane (Figure 3A). Additionally, we observed changes in fluorescence of intracellular Ca^2+^ under inverted fluorescence microscopy (Figure 3B). The rise in intracellular calcium is reduced when the cells were incubated with PPADS or suramin (50 μM) for 30 min prior to treatment with ATP (Figure 3C).

### Effects of extracellular ATP on the levels of p65 and OPN in NPC cells

**Figure 4:**
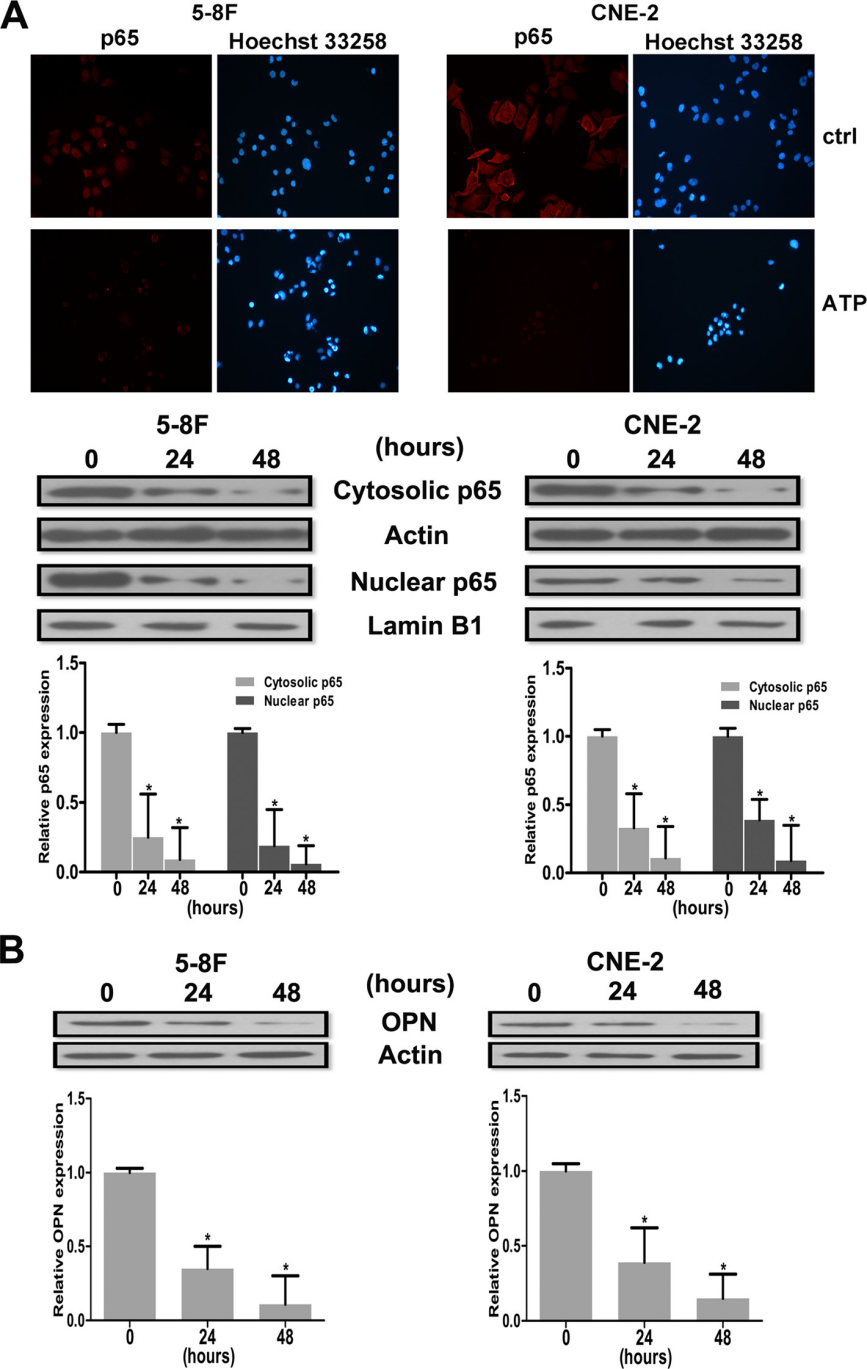
**The effects of extracellular ATP on the expression of p65 and OPN in NPC cells. A**: 5-8 F or CNE-2 cells were incubated with ATP (100 μM), and western blotting and immunofluorescence were used to detect the expression of p65. A notable decrease in p65 in the nucleus of 5-8 F and CNE-2 cells treated with ATP was found. The intensity of the bands was quantified. **B**: 5-8 F or CNE-2 cells were incubated with ATP (100 μM) for the indicated time, and OPN expression was detected by western blotting, with quantification of the band intensity. Similar to the situation for p65 in NPC cells, ATP significantly downregulated OPN expression in 5-8 F and CNE-2 cells. Statistical significance between the control and treated conditions: *P < 0.05.

We detected the expression of p65 and OPN in NPC cells using immunofluorescence and western blotting assays. NPC cells were treated with ATP (100 μM) for the indicated time, and the cells were then subjected to immunofluorescence and western blotting, as shown in Figure 4A. ATP was found to significantly decrease the expression of cytosolic and nuclear p65 in both the 5-8 F and CNE-2 cells, similar to the situation for p65, and ATP also decreased the expression of OPN in the two NPC cell lines (Figure 4B).

### Vector-based RNAi decreases P2Y2 expression

**Figure 5:**
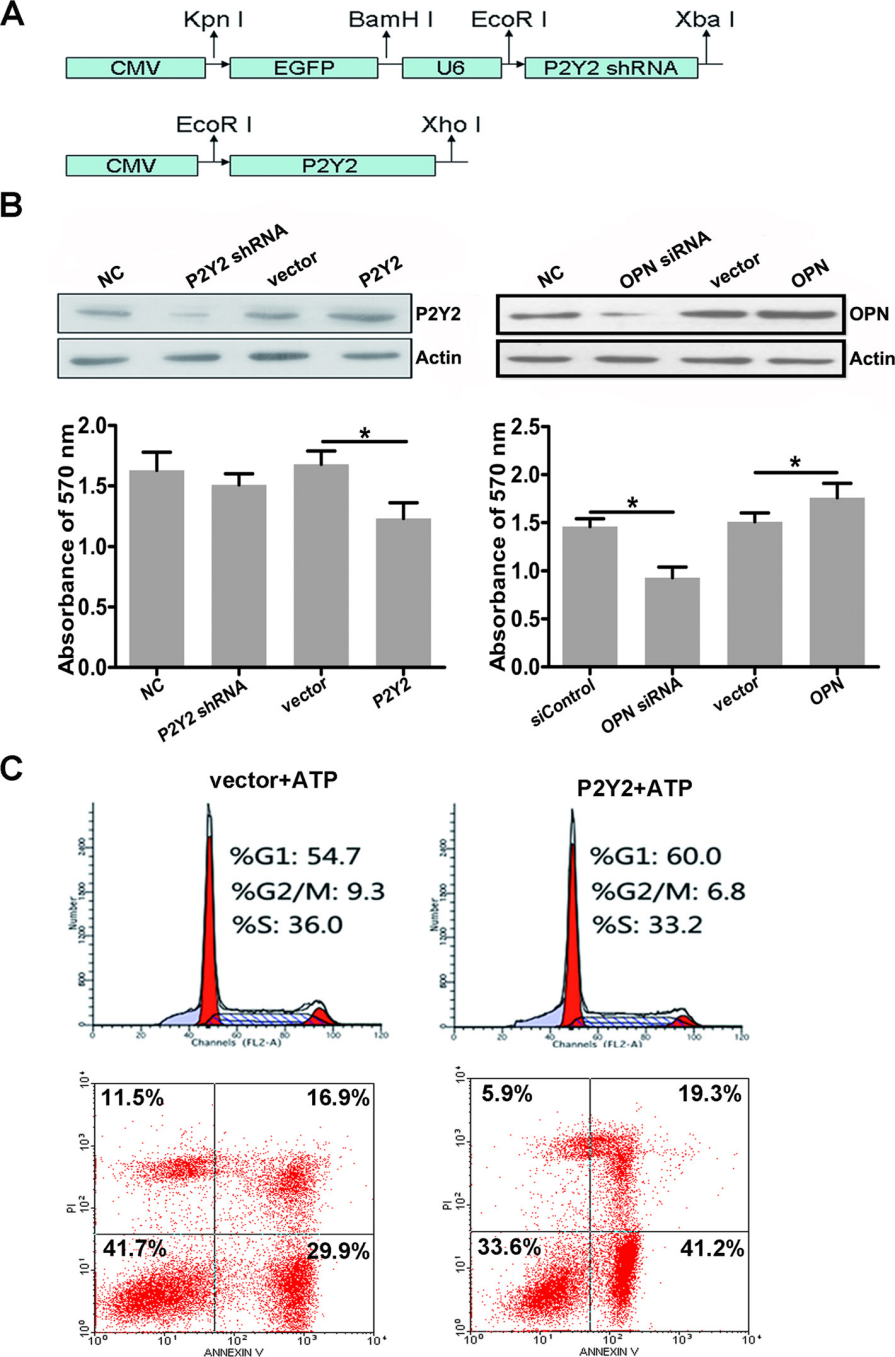
**The role of P2Y2 in mediating the effects of ATP on NPC cells. A**: Schematic representation of the constructed vectors. CMV, CMV promoter; EGFP, enhanced green fluorescent protein gene; U6, U6 promoter. **B**: P2Y2 and OPN regulate the growth inhibitory effects of ATP on NPC cells. The levels of P2Y2 and OPN were significantly decreased after transfection with the shRNA expressing vector or siRNA. P2Y2 overexpression enhanced the growth inhibitory effect of ATP on NPC cells, whereas OPN overexpression reversed this effect. There were statistically significant differences between the control and treated conditions: *P < 0.05. **C**: The role of P2Y2 expression in the effects of ATP on the cell cycle and apoptosis of CNE-2 cells. P2Y2 expression increased the percentage of G1-phase cells and decreased the proportion of G2/M-phase cells. The overexpression of P2Y2 increased the level of apoptotic cells in ATP treated cells.

As there is scant literature describing vector-based RNAi of P2Y2 expression, we selected an effective vector to downregulate the level of P2Y2. Western blotting (Figure 5B) revealed that the CNE-2 cells transfected with P2Y2 shRNA exhibited a much weaker band compared to the control group. This result indicated that the constructed P2Y2 shRNA-expressing vector could effectively downregulate the protein level of P2Y2. In contrast, pcDNA3.1-P2Y2 upregulated the protein level of P2Y2 in CNE-2 cells.

### Effects of P2Y2 and OPN on CNE-2 cell chemosensitivity to ATP

To examine the role of P2Y2 and OPN in the effect of ATP on the proliferation of CNE-2 cells, the cells were transfected with constructed plasmids or siRNA for 24 h and then cultured with ATP (100 μM) for another 48 h. An MTT assay was used to evaluate cell viability after treatment with ATP. As shown in Figure 5B, we found that OPN downregulation increased the sensitivity of CNE-2 cells to ATP, whereas OPN overexpression resulted in a higher cell viability compared to the corresponding control group. OPN overexpression itself showed no obvious effect on the viability of CNE-2 cells [23]. We further found that the upregulation of P2Y2 could decrease CNE-2 cell viability, with P2Y2 downregulation having no obvious influence on CNE-2 cell viability. These results indicated that P2Y2 and OPN participate in the effect of ATP on the proliferation of CNE-2 cells.

### Effects of P2Y2 expression on CNE-2 cell cycle and apoptosis

The cell cycle distribution was analyzed by flow cytometry after the cells were transfected with the P2Y2 expression vector for 24 h and treated with 100 μM ATP for another 48 h. As shown in Figure 5C, the proportion of pcDNA3.1-P2Y2-transfected cells in G2/M phase decreased (9.3% to 6.8%), whereas those in G1 phase increased (54.7% to 60%) compared to the control group. To assess apoptosis, cells were transfected with the P2Y2 expression vector for 24 h and were then treated with 100 μM ATP for another 48 h before analysis by flow cytometry. The results indicated that overexpression of P2Y2 increased the apoptosis of CNE-2 cells (Figure 5C).

### Effect of P2Y2 on the expression of p65 and OPN in NPC cells

**Figure 6:**
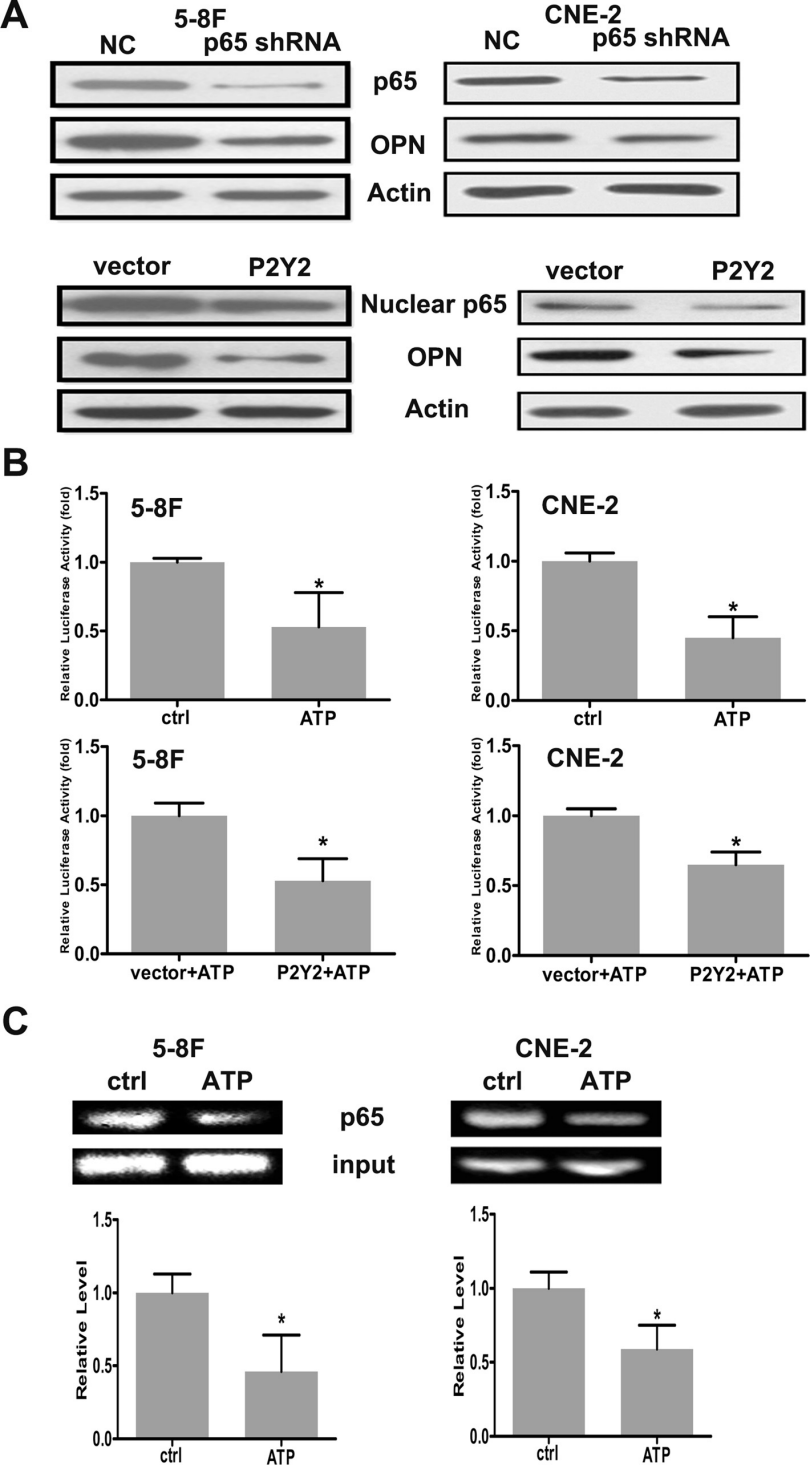
**Effects of ATP and P2Y2 on the regulation of OPN by p65. A**: Overexpression of P2Y2 downregulated the levels of p65 and OPN, and p65 knockdown with shRNA decreased the level of OPN in 5-8 F and CNE-2 cells. **B**: ATP and P2Y2 reduced the transcriptional activity of OPN promoter. **C**: ATP lowered the binding of p65 to the promoter region of OPN in 5-8 F and CNE-2 cells. The data represent the means ± SD of 3 independent experiments. Statistical significance between the control and treated conditions: *P < 0.05 (100 μM).

As P2Y2 might be involved in the ATP-induced downregulation of p65 and OPN, we further examined the effect of P2Y2 on the level of p65 and OPN by western blotting. As shown in Figure 6A, after 5-8 F and CNE-2 cell transfection of the constructed plasmids for 72 h, the levels of p65 and OPN were decreased in the P2Y2-overexpression group. It has been reported that p65 can upregulate the expression of OPN in some tumor cells [24], and we confirmed the potential regulation of OPN by p65 and the effects of ATP on the transcriptional activity of p65 toward OPN in 5-8 F and CNE-2 cells. We found that OPN decreased when the 5-8 F and CNE-2 cells were transfected with the p65 shRNA-expressing vector, and a luciferase activity assay indicated that ATP and its P2Y2 receptor could decrease the transcriptional activity of OPN promoter in 5-8 F and CNE-2 cells (Figure 6B). Using a ChIP assay, we further found that ATP could lower the binding of p65 to potential sites in the OPN promoter region (Figure 6C). Accordingly, ATP might exert its inhibitory effects in NPC cells, at least in part, via P2Y2, p65, and OPN.

### P-AKT is involved with OPN in the chemosensitivity of CNE-2 cells to ATP

**Figure 7:**
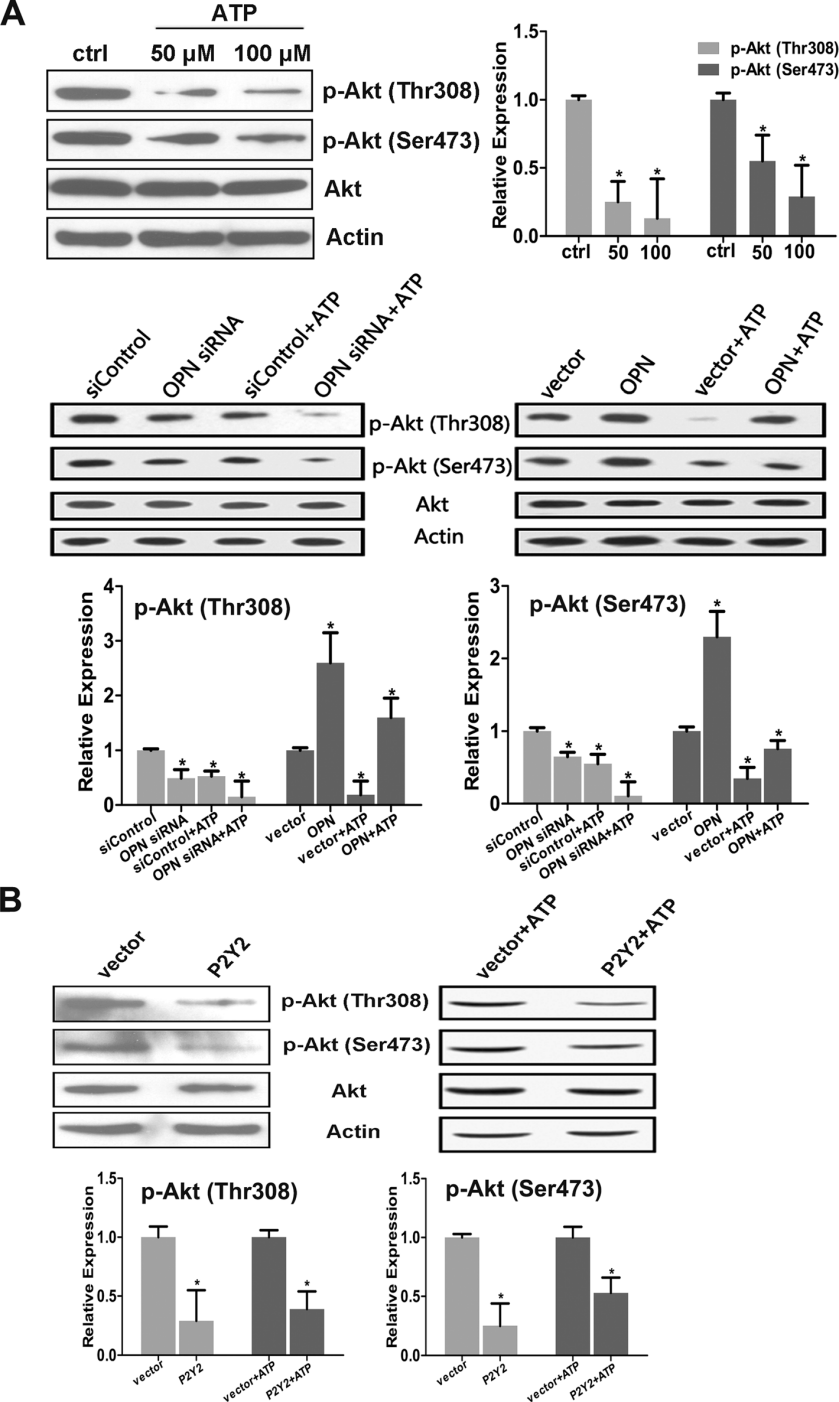
**P-AKT was involved in the effects of ATP and P2Y2 in NPC cells. A**: The Akt pathway was involved in the effect of ATP on NPC cells. ATP decreased the level of p-Akt in CNE-2 cells, and OPN downregulation decreased the level of p-Akt and enhanced the extent of the downregulation of p-Akt by ATP. The overexpression of OPN increased the level of p-Akt and mitigated the effect of ATP on the p-Akt level. **B**: The overexpression of P2Y2 could decrease the p-AKT level in CNE-2 cells. The level of p-Akt was detected by western blotting, with quantification of the band intensity. Statistical significance between the control and treated conditions: *P < 0.05.

It has been reported OPN can influence the level of p-AKT in some tumor cells, and vice versa [24-26]; however, the situation in NPC cells is still unknown. We detected the relationship between p-AKT and OPN on the effects of ATP after CNE-2 cell transfection with OPN the siRNA or OPN expression vector. The cells were further incubated with ATP (100 μM) for 48 h after 24 h of transfection, and western blotting assays were performed. As shown in Figure 7A, OPN downregulation decreased the level of p-AKT (both Thr308 and Ser473), whereas OPN upregulation had the opposite effect of increasing p-AKT (both Thr308 and Ser473). ATP alone also decreased the level of p-AKT in a dose-dependent manner, which was facilitated by OPN downregulation. We also verified that P2Y2 overexpression further increased the effect of ATP on the level of p-AKT in CNE-2 cells (Figure 7B).

## Discussion

Our results show that extracellular ATP decreased the viability and inhibited the migration of 5-8 F and CNE-2 cells. Notably, ATP-induced cell cycle arrest in S phase was significant in these NPC cells. Given that certain chemotherapeutic drugs are more cytotoxic to cells arrested in S phase [27,28], the recruitment of cancer cells to S phase by ATP might sensitize poorly differentiated NPC cells to these drugs. Apoptosis was also induced in the NPC cells treated with ATP. P2 receptors might be involved in these effects. Because P2Y2 receptor is the main subtype for ATP, P2Y2 might participate in the effects of ATP on NPC cells. In the present study, P2Y2 promoted the growth inhibition effects of ATP on NPC cells, similar to the situation in some other tumor cells [4]. However, some reports have shown that ATP can stimulate the proliferation of cancer cells [29,30], though the underlying mechanism is unclear. For the first time in NPC cells, our study showed that extracellular ATP inhibits the proliferation and migration of NPC cells via the downregulation of p65 and OPN. A recent report provided evidence that ATP could inhibit the migration of NPC cells (CNE2Z) through the blockage of volume-activated chloride channels [31], which is opposite to the situation in SMCs [21]. OPN, which was detected in both 5-8 F and CNE-2 cells by western blotting, has been studied extensively in several tumor models and was found to be involved in the regulation of several signal transduction pathways and factors, including the AKT pathway [24-26,32]. One recent report also provided evidence that OPN could regulate the growth of NPC cells [23]. Here, we report for the first time that both OPN and ATP can affect the level of p-AKT in NPC cells, and our results also indicate that the AKT pathway might be involved in the effects of ATP and OPN on NPC cells. As the inhibition of OPN expression can result in a decrease in the metastatic potential of tumor cells, these findings may therefore be of great benefit for patient prognosis. Indeed, a higher level of OPN was detected in the serum of NPC patients, and a higher level of OPN was shown to decrease the sensitivity of NPC cells to radiotherapy [33,34]. Given that radiotherapy is the main therapy for NPC, the downregulation of OPN by ATP might sensitize poorly differentiated NPC cells to radiotherapy and decrease the metastatic potential of these cells. Clearly, the effect of ATP on OPN expression in other tumor cells needs to be studied further.

Similar to many other purinergic receptors, the function of P2Y2 in tumor cells remains poorly understood. Some reports have proposed a possible function of P2Y2 in tumor cells via changes in ion flux, and new functional purinergic receptors are constantly being reported. In our study, we constructed plasmids to specifically regulate the P2Y2 level and then investigated its potential function. We showed that P2Y2 is involved in the effect of ATP on NPC cells via p65 and OPN and verified that it exhibited growth inhibitory effects on tumor cells on its own, results that are apparently contradictory to the reports suggesting that P2Y2 is involved in the promotion of proliferation [29,30]. ATP and its receptor P2Y2 might exert opposite effects on migration in different cell types: ATP and P2Y2 were found to stimulate the migration of corneal epithelial cells but inhibit human keratinocyte spread and migration [35,36]. In the present study, ATP exerted an inhibitory effect on NPC cell motility, whereas P2Y2 itself showed no influence on motility. We also used RT-PCR was used to detect the expression of purinergic receptors in NPC cells, and future studies will be aimed at investigating the function of these receptors in tumor cells. The expression of P2Y2 receptors in 5-8 F and CNE-2 cells was also examined by immunofluorescence microscopy.

It is well known that p65 signaling has important roles in carcinogenesis, cancer development and progression [37]. In addition, p65 signaling was involved in the effects of chemotherapeutic agents [38], and it might be the attractive targets for the development of new anti-cancer drugs [39]. The relationship between purinergic signaling and p65 signaling in cancer cells is still unclear. One recent report indicated that ATP could inhibit the proliferation of endothelial progenitor cells via inhibiting TLR4 activation induced phosphorylation of p65 [40]. In this study, we provided preliminary data about the effects of ATP and P2Y2 on p65 signaling in NPC cells.

In conclusion, the present results show that extracellular ATP inhibits the growth and migration of NPC cell lines, and some of these effects are mediated by the downregulation of p65 and OPN via P2Y2. Therefore, ATP could be a promising agent serving as an adjuvant in the treatment of NPC. P2Y2 and OPN might be potential targets of gene therapy, though further research in NPC cells is needed.

## Competing interests

The authors have no competing interest to declare.

## Authors’ contributions

GY and FJZ conceived and designed the experiments. GY, SHZ and YLZ performed the experiments. QMZ and SP analyzed the data. TZ and CFY supported the experiments and helped to draft the manuscript. ZYZ performed the statistical analysis. GY wrote the manuscript. All authors read and approved the final manuscript.
